# Hierarchical structure and modules in the *Escherichia coli *transcriptional regulatory network revealed by a new top-down approach

**DOI:** 10.1186/1471-2105-5-199

**Published:** 2004-12-16

**Authors:** Hong-Wu Ma, Jan Buer, An-Ping Zeng

**Affiliations:** 1 Department of Genome Analysis, GBF – German Research Center for Biotechnology, Mascheroder Weg 1, 38124 Braunschweig, Germany; 2 Department of Mucosal Immunity, GBF – German Research Center for Biotechnology, Mascheroder Weg 1, 38124 Braunschweig, Germany; 3 Medical Microbiology and Hospital Hygiene, Medical School Hannover, Carl-Neuberg-Str. 1, 30625 Hannover, Germany

## Abstract

**Background:**

Cellular functions are coordinately carried out by groups of genes forming functional modules. Identifying such modules in the transcriptional regulatory network (TRN) of organisms is important for understanding the structure and function of these fundamental cellular networks and essential for the emerging modular biology. So far, the global connectivity structure of TRN has not been well studied and consequently not applied for the identification of functional modules. Moreover, network motifs such as feed forward loop are recently proposed to be basic building blocks of TRN. However, their relationship to functional modules is not clear.

**Results:**

In this work we proposed a top-down approach to identify modules in the TRN of *E. coli*. By studying the global connectivity structure of the regulatory network, we first revealed a five-layer hierarchical structure in which all the regulatory relationships are downward. Based on this regulatory hierarchy, we developed a new method to decompose the regulatory network into functional modules and to identify global regulators governing multiple modules. As a result, 10 global regulators and 39 modules were identified and shown to have well defined functions. We then investigated the distribution and composition of the two basic network motifs (feed forward loop and bi-fan motif) in the hierarchical structure of TRN. We found that most of these network motifs include global regulators, indicating that these motifs are not basic building blocks of modules since modules should not contain global regulators.

**Conclusion:**

The transcriptional regulatory network of *E. coli *possesses a multi-layer hierarchical modular structure without feedback regulation at transcription level. This hierarchical structure builds the basis for a new and simple decomposition method which is suitable for the identification of functional modules and global regulators in the transcriptional regulatory network of *E. coli*. Analysis of the distribution of feed forward loops and bi-fan motifs in the hierarchical structure suggests that these network motifs are not elementary building blocks of functional modules in the transcriptional regulatory network of *E. coli*.

## Background

Genome sequencing and high-throughput technologies of functional genomics generate a huge amount of information about cellular components and their functions in an unprecedented pace. These advances make it possible to reconstruct large scale biological networks (metabolism, gene regulation, signal transduction, protein-protein interaction etc.) at a whole cell level [1-4]. One of the key issues in the contemporary genomic biology is to understand the structure and function of these cellular networks at different molecular levels. Among them, the transcriptional regulatory network (TRN) plays a central role in cellular function because it regulates gene expression and metabolism and is often the final step of signal transduction [5,6]. Genome scale TRNs have been reconstructed for well studied organisms such as *Escherichia coli *and *Saccharomyces cerevisiae *[4,5,7,8]. Recent studies of TRNs have been concentrated on the topological structure and its correlation with gene expression data from microarray experiments, the evolutionary relationship between regulators, the network motifs and the global regulators in the network etc [7-15]. Network motifs are regarded as the basic building blocks of complex networks [16,17]. Feed forward loop (FF loop) and Bi-fan motif were found to be the two most important network motifs in TRN [7]. In a recent study, Dobrin et al. [9] reported that the motifs in *E. coli *TRN aggregated into homologous motif clusters that largely overlapped with known biological functions and further formed a giant motif supercluster which comprised about half of the nodes in the giant component of the whole network. This study provided interesting information for understanding the organization principle of regulatory networks. A different approach for studying network organization is the so called "top-down view"[18]. It starts from the whole network structure and identifies subsystems or modules by network decomposition. It is generally recognized that most cellular functions are coordinately carried out by groups of genes forming functional modules [19-25]. The identification of modules is thus an essential step for obtaining any testable biological hypotheses from the network structure. Several methods have been proposed to detect modules in metabolic networks and protein-protein interaction networks based on the topology of the network [21,26-30]. As shown in our recent work [27] the global connectivity structure of metabolic network was useful for a more reasonable decomposition of it into functional modules. However, the global structure of TRN has been so far not taken into account in its decomposition. In fact, little is known about the global connectivity structure of TRN.

In this work we demonstrated the applicability of a top-down approach for the identification of functional modules in TRN with the well established transcriptional regulatory network of *E. coli *as an example. For this purpose we first showed an uncovered global hierarchical structure. Global regulators and modules with clearly defined functions were then identified by a new network decomposition method based on the hierarchical structure. We further investigated the distribution of the two basic network motifs, feed forward loop and bi-fan motif, in the network hierarchical structure and examined their relationship to functional modules.

## Results and discussion

### The hierarchical structure of regulatory network

**Figure 1:**
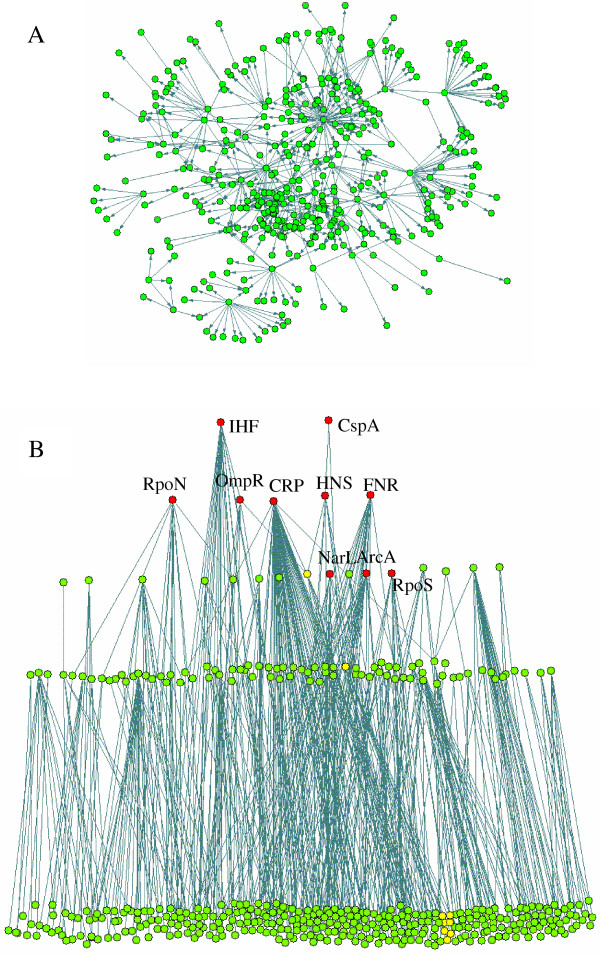
**Hierarchical structure of *E. coli *transcriptional regulatory network. **A: The original unorganized network. B: the hierarchical regulation structure in which all the regulatory links are downward. Nodes in the graph are operons. Links show the transcriptional regulatory relationships. The global regulators found in this work are shown in red. The yellow marked nodes are operons in the longest regulatory pathway related with flagella motility.

The transcriptional regulatory network of *E. coli *considered in this work is based on RegulonDB [5] and complemented by Shen-Orr et al [7]. It consists of 413 nodes and 576 links as shown in Fig. 1A. To investigate the network global connectivity, we calculated the weakly connected components and the strongly connected components in the network using the software Pajek [31]. A subset of nodes in a network is called a weakly connected component (WCC) if from every node of the subset all the other nodes belonging to the same subset can be reached when ignoring the direction of the links. If the direction is considered such a fully connected subset is then called a strongly connected component (SCC). We found 28 WCCs in the network. The largest one (the so-called giant component) consists of 325 operons which accounts for more than three quarters of the whole network. Among the other WCCs 20 of them contain only two operons and only 4 WCCs contain more than five operons. The existence of the giant component in TRN is similar to that found previously in metabolic networks[32]. However, different from metabolic networks we find that there is no SCC in the TRN of *E. coli*. This means that there are no regulatory cycles (e.g. gene *A *regulates gene *B *and gene *B *also regulates gene *A *through another path) in the TRN of *E. coli*. This result implies an acyclic structure of the *E. coli *TRN in which the nodes can be placed in different layers according to their depth. To identify such a hierachical structure we rearranged the operons in the following way: (1) operons which do not code for transcription factors (TFs) or code for a TF which only regulates its own expression (auto-regulatory loop) were assigned to layer 1 (the lowest layer); (2) then we removed all the operons in layer 1 and from the remaining network identified TFs which do not regulate other operons and assigned the corresponding operons in layer 2; (3) we repeated step 2 to remove nodes which have been assigned to a layer and identified a new layer until all the operons were assigned to different layers. As a result, a five layer hierarchical structure was uncovered as shown in Fig. 1B. All the regulatory links in this graph are downward and there is no link between operons in the same layer (except the auto-regulatory loops).

The multi-layer hierarchical structure of the *E. coli *TRN implies that no feed back regulation exists at transcription level. We noticed that Shen-Orr et al have also reported that there was no feed back loop in the *E. coli *regulatory network [7]. We further examined the yeast regulatory network proposed by Guelzim et al [8] and found it also has a similar hierarchical structure (result not shown). This gives rise to the question why the transcriptional regulatory networks of these organisms possess such an acyclic hierarchical structure. A possible explanation is that the interactions in TRN are interactions between proteins and DNAs. Therefore, a regulated gene must has been transcribed and translated into its protein product (which is eventually further modified by cofactor binding) to make a feedback interaction between it and its regulator gene possible. The well studied *lac *operon may be used as an example to further illustrate this point. *Lac *operon is not expressed unless lactose is available for the cell because it is repressed by the *lac *repressor. *Lac *repressor (the protein but not the gene) is the control element of the system. Its existence (expression) is necessary for the cell to properly response to environmental changes (i.e. the presence and absence of lactose). Therefore, for cells to quickly and properly response to changes of environmental conditions it is of advantage to keep a set of proteins expressed in all conditions and through them to regulate the expression of other genes in a hierarchical way. Feedback control of gene expression may be mainly through other interactions (e.g. metabolite and protein interaction) rather than through transcriptional interactions between proteins and genes. In fact, many transcription factors can bind small molecules to gain or loss their ability to bind DNA.

The five-layer hierarchy shown in Fig. 1B does not necessarily mean that TFs at the top layer require 4 steps to regulate operons at the bottom layer. In fact, many operons at the bottom layer are directly regulated by top layer TFs. Among the 717 linked pairs of operons, 516 are directly connected. The average path length of the network is only 1.36, suggesting a fast and efficient response of cells to environment perturbations in general. The longest regulation path in the network is *IHF *→ *OmpR *→ *FlhDC *→ *FliA *and further to seven operons (marked yellow in Fig. 1B) related to flagella motility. The finding that there is no short-cut between these regulators and the regulated operons is unexpected. Regulatory relationships may exist between them but are not yet identified. Actually five operons that are regulated by *FliA *are also directly regulated by *FlhDC*, resulting in a shorter path between the upper layer regulators (*FlhDC*) and these operons.

### Network decomposition, global regulators and modules

Based on the uncovered hierarchical organization structure we propose a new method to identify functional modules in TRN. As discussed above, there is a giant weakly connected component in the whole TRN of *E. coli*. We find that the giant component preserves the five layer hierarchical structure of the whole network. It also includes the single large motif super cluster found by Dorbin et al. [9] and thus preserves most of the network motifs in the whole network. Therefore, in the subsequent steps we focus on this giant component to present a new method to identify global regulators and modules in the network.

**Figure 2:**
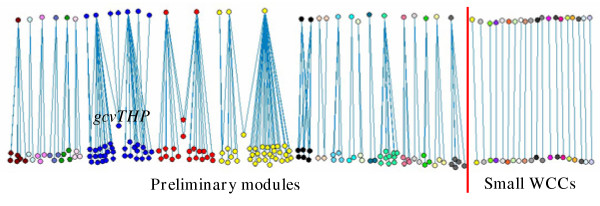
**Preliminary modules in the reduced transcriptional regulatory network of *E. coli. ***All the operons in the top three layers (Fig. 1b) and operons which are regulated only by them were removed to reduce the network. The weakly connected components of the reduced network were calculated and shown in different colors. Only WCCs which contain at least three nodes were considered as preliminary modules. The small WCCs which contain only two nodes were grouped at upper regulation level. The three largest WCCs were split into two preliminary modules by investigating their connectivity.

**Figure 3:**
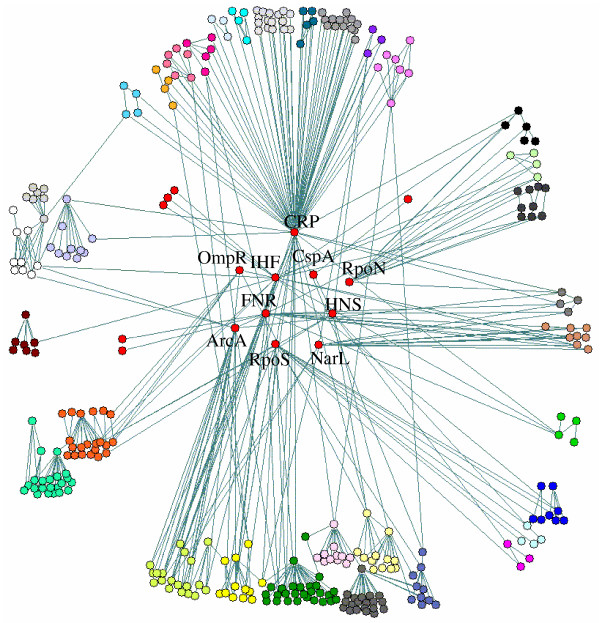
**Functional modules in the transcriptional regulatory network of *E. coli*. **Operons in different modules are shown in different colors. The ten global regulators form the core part of the network. The periphery modules are connected mainly through the global regulators. Depending on the connectivity between the modules and their connectivity to the global regulators, these modules can be further grouped to larger modules at a higher level.

First we removed all the operons in the top three layers and the operons which are regulated only by them, resulting in a network with 221 nodes and 186 links as shown in Fig. 2. We found 41 weakly connected components in this reduced network (shown in different colors in Fig. 2). In contrast to the whole network, there is no giant component in the reduced network. The three largest components contain 38, 36 and 21 operons respectively (the nodes in blue, yellow and red respectively). It can be seen from Fig. 2 that these three components are connected by only one or two nodes. Therefore we can decompose them into two relatively independent parts by cutting through these nodes. For example, the largest component was separated by cutting *gcvTHP *which codes enzymes for glycine cleavage. We checked the function of the operons in the two separated parts by using EcoGene database [33]. The left part of this WCC is mainly for purine synthesis and the right part for amino acid uptake. All the other WCCs are very small with less than 12 operons. Most of them are regulated by only one regulator and thus they are in the same regulon. The functions of the operons in these WCCs are closely related. Therefore, we considered the WCCs (the two split parts for the three largest WCCs) which contain at least three nodes as preliminary modules in the network. Altogether 24 preliminary modules were obtained. The 20 small WCCs which contain only two nodes may be regulated by the same regulators at upper layers and thus can be grouped in the next organization level. In the next step, we extended the 24 preliminary modules by moving upward to include the regulators at the third, fourth and fifth layers consecutively and their regulated operons. Each of the regulator was investigated to find its linked preliminary modules and the number of links between them. The regulator was then classified into the module with the most connections. If a regulator has more links with operons which have not been assigned to any preliminary module than with any preliminary module, it formed a new module together with its regulated operons. In this way, the many small two-node components in the low hierarchy level can be grouped to form new modules. On the other hand, the regulators that regulate operons in three or more preliminary modules were regarded as global regulators and not assigned to any module. Using this method, 10 global regulators (Table 1 and Fig. 1B) and 33 modules (Table 2 and Fig. 3) are identified. In addition, 6 modules found from the small WCCs of the whole network which contain at least three operons are also included in Table 2. In Fig. 3 we place the ten global regulators in the central part, whereas the 33 modules are in the periphery part around them. We can see that the periphery modules are connected mainly through the global regulators.

**Table 1:** Global regulators and their regulated operons and functions in the regulatory network of *E. coli*.

Global regulator	directly regulated Operons	Total regulated operons	Modules regulated	Function
*IHF*	21	39	15	integration host factor
*CspA*	2	24	5	Cold shock protein
*CRP*	72	112	21	cAMP receptor protein
*FNR*	22	38	16	anaerobic regulator, regulatory gene for nitrite and nitrate reductases, fumarate reductase
*HNS*	7	22	5	DNA-binding global regulator; involved in chromosome organization; preferentially binds bent DNA
*OmpR*	6	20	3	Response regulator for osmoregulation; regulates production of membrane proteins
*RpoN*	12	17	4	RNA polymerase sigma 54 subunit
*RpoS*	14	24	8	stationary phase sigma factor
*ArcA*	20	21	6	Response regulator protein represses aerobic genes under anaerobic growth conditions and activates some anaerobic genes
*NarL*	13	15	5	Two-component regulator protein for nitrate/nitrite response

**Table 2:** Functional investigation of modules identified.

index	Operons included	Biological function description
1	*aceBAK, acs, adhE, fruBKA, fruR, icdA, iclMR, mlc, ppsA, ptsG, ptsHI_crr, pykF*	Hexose PTS transport system, PEP generation, Acetate usage, glyoxylate shunt
2	*acnA, fpr, fumC, marRAB, nfo, sodA, soxR, soxS, zwf*	Oxidative stress response
3	*ada_alkB, aidB, alkA, ahpCF, dps, gorA, katG, oxyR*	Oxidative stress response, Alkylation
4	*alaWX, aldB, argU, argW, argX_hisR_leuT_proM, aspV, dnaA, leuQPV, leuX, lysT_valT_lysW, metT_leuW_glnUW_metU_glnVX, metY_yhbC_nusA_infB, nrdAB, pdhR_aceEF_lpdA, pheU, pheV, proK, proL, proP, sdhCDAB_b0725_sucABCD, serT, serX, thrU_tyrU_glyT_thrT, thrW, tyrTV, valUXY_lysV, yhdG_fis*	rRNA, tRNA genes, DNA synthesis system, pyruvate dehydrogenase and ketoglutarate dehydrogenase system
5	*araBAD, araC, araE, araFGH, araJ*	Arabinose uptake and usage
6	*argCBH, argD, argE, argF, argI, argR, carAB*	Arginine usage, urea cycle
7	*caiF, caiTABCDE, fixABCX*	Carnitine usage
8	*clpP, dnaKJ, grpE, hflB, htpG, htpY, ibpAB, lon, mopA, mopB, rpoH*	Heat shock response
9	*codBA, cvpA_purF_ubiX, glnB, glyA, guaBA, metA, metH, metR, prsA, purC, purEK, purHD, purL, purMN, purR, pyrC, pyrD, speA, ycfC_purB, metC, metF, metJ*	Purine synthesis, purine and pyrimidine salvage pathway, methionine synthesis
10	*cpxAR, cpxP, dsbA, ecfI, htrA, motABcheAW, ppiA, skp_lpxDA_fabZ, tsr, xprB_dsbC_recJ*	Stress response, Conjugative plasmid expression, cell motility and Chemotaxis
11	*dctA, dcuB_fumB, frdABCD, yjdHG*	C4 dicarboxylate uptake
12	*edd_eda, gntKU, gntR, gntT*	Gluconate usage, ED pathway
13	*csgBA, csgDEFG, envY_ompT, evgA, gcvA, gcvR, gcvTHP, gltBDF, ilvIH, kbl_tdh, livJ, livKHMGF, lrp, lysU, ompC, ompF, oppABCDF, osmC, sdaA, serA, stpA*	Amino acid uptake and usage
14	*fdhF, fhlA, hycABCDEFGH, hypABCDE*	Formate hydrogenlyase system
15	*flgAMN, flgBCDEFGHIJ, flgKL, flgMN, flhBAE, flhDC, fliAZY, fliC, fliDST, fliE, fliFGHIJK, fliLMNOPQR, tarTapcheRBYZ*	Flagella motility system
16	*ftsQAZ, rcsAB, wza_wzb_b2060_wcaA_wcaB*	Capsule synthesis, cell division
17	*gdhA, glnALG, glnHPQ, nac, putAP*	Glutamine and proline utilization
18	*glmUS, manXYZ, nagBACD, nagE*	Glucosamine, mannose utilization
19	*glpACB, glpD, glpFK, glpR, glpTQ*	Glycerol phosphate utilization
20	*lysA, lysR, tdcABCDEFG, tdcR*	Serine, threonine usage
21	*malEFG, malK_lamB_malM, malPQ, malS, malT, malZ*	Maltose utilization
22	*rhaBAD, rhaSR, rhaT*	Rhamnose utilization
23	*appCBA, appY, betIBA, betT, cydAB, cyoABCDE, fadBA, focA_pflB, fumA, glcC, glcDEFGB, gltA, lctPRD, mdh, nuoABCEFGHIJKLMN, fabA, fadL, fadR, uspA*	Oxidative phosphorylation, Glycolate, lactose utilization, fatty acid degradation
24	*cytR, deoCABD, deoR, nupC, nupG, tsx, udp*	Nucleosides uptake and usage
25	*cirA, entCEBA, fecABCDE, fecIR, fepA_entD, fepB, fepDGC, fhuACDB, fur, tonB*	Iron uptake system
26	*galETKM, galR, galS, mglBAC*	Galactose uptake and usage
27	*dmsABC, fdnGHI, narGHJI, narK, nirBDC_cysG, nrfABCDEFG, torCAD, torR*	Nitrogen metabolism, Nitrate and nitrite reductase,
28	*narZYWV, nhaA, nhaR, osmY*	intracellular pH regulation
29	*aslB, inaA, mdlA, rob, ybaO, ybiS, yfhD*	Stress response
30	*cutC, dapA_nlpB_purA, ecfABC, ecfD, ecfF, ecfG, ecfH, ecfJ, ecfK, ecfLM, fkpA, ksgA_epaG_epaH, lpxDA_fabZ, mdoGH, nlpB_purA, ostA_surA_pdxA, rfaDFCL, rfbO, rpoE_rseABC, uppS_cdsA_ecfE*	RpoE regulated stress response, lipopolysaccharide synthesis
31	*ansB, cpdB, cyaA, dadAX, epd_pgk, glgCAP, glgS, ivbL_ilvBN, ompA, speC, srlAEBD_gutM_srlR_gutQ, tnaLAB, ubiG, yhfA*	Sorbitol and Glycogen metabolism
32	*atoC, atoB, hydHG, hypA, pspABCDE, pspF, rtcAB, rtcR, zraP*	Phage shock protein, Zn-resistence system, Acetoacetate metabolism
33	*dsdC, dsdXA, ebgAC, ebgR, fucAO, fucPIKUR, lacI, lacZYA, malI, malXY, melAB, melR, uhpA, uhpT, yiaJ, yiaKLMNOPQRS*	Lactose, maltose, fucose, dehydroascorbate, xylulose, melibiose transport and metabolism
34	*aroF_tyrA, aroG, aroH, aroL_yaiA_aroM, aroP, mtr, trpLEDCBA, trpR, tyrP, tyrR*	Aromatic amino acid synthesis
35	*bioA, bioBFCD, birA_murA*	Biotin synthesis
36	*cbl, cysB, cysDNC, cysJIH, cysK, cysPUWAM, ssuEADCB, tauABCD*	Sulfur metabolism, cysteine synthesis, Taurine utilization
37	*exuR, exuT, uidR, uidABC, uxaCA, uxuABR*	Utilization of hexUronide
38	*lexA_dinF, polB, recA, recN, rpsU_dnaG_rpoD, ssb, sulA, umuDC, uvrA, uvrB, uvrC, uvrD*	DNA recombination and repair, UV resistent
39	*phnCDE_f73_phnFGHIJKLMNOP, phoA, phoBR, phoE, pstSCAB_phoU*	Phosphate metabolism

To investigate if the modules identified from anaylisis of the network structure are really functionally related, we checked the functions of the genes in the individual modules by using database EcoGene [33]. Most genes in the same module turned out to have closely related biological function. Thus, we can assign clearly defined functions for most of the modules. However, there are also several modules which include operons that are seemingly functionally not closely related. For example, there are also several operons for acetate usage in module 1 (Table 2) besides the operons for the PTS sugar transport system. One of the acetate usage operons *aceBAK *is also repressed by *FruR*, the regulator for fructose uptake. This makes the cell not to use acetate as a substrate in the presence of fructose. Therefore, the two different pathways are actually functionally related from a regulation viewpoint. The other three modules (module 4, 23 and 32) which include operons with different functions are actually linked by certain global regulators (*fis, arcA *and *rpoN *respectively). They are not connected by any local regulators with specific functions. Thus, it is not strange that they are not closely functionally related. One reason for this problem is probably the information incompleteness of the network. The regulatory network considered in this work contains only about twenty percent of the genes in the *E. coli *genome. With more and more information available we can include more interactions and genes in the network to obtain more reasonable modules by structural analysis. Identifying these functional modules can help us to gain a general view of the function (or ability) of organisms. Furthermore, we can compare these structure based modules with modules from hierarchical classification results of microarray experiments to find unknown regulatory relationships.

We compared the ten global regulators with those found in three previous studies by considering the number of directly or indirectly regulated genes (operons) and their structure and function diversity [7,10,11]. Five of them (*CRP, IHF, FNR, HNS, ArcA*) have been identified in all the three studies. The other five regulators have also been recognized as global in either one or two of the three studies. Our definition of global regulators is directly linked to the identification of functional modules. Modules are sets of genes with closely related function. An important criterion for a regulator to be regarded as global is that it regulates genes with diverse but concerted functions. Therefore, determination of global regulators by the number of regulated modules is more reasonable than that solely by the number of genes or operons. From Fig. 3 we can see that the number of links among the modules is far less than that between the global regulators and the modules. This indicates that the global regulators introduce the major cross-talks between modules and link them together to form the whole network. Therefore, breaking the links through the global regulators can help to identify the true modules as shown in this work.

### Network motifs and motif clusters

To investigate if network motifs, which are considered to be the elementary building blocks of the whole network [17], are basic building blocks of modules and if motif clusters are generally equivalent to functional modules, we calculated the feed forward loops (FF loops) in the TRN of *E. coli*. In agreement with the results of Dobrin et al [9], the 42 FF loops in the network aggregate to seven homologous motif clusters (see Additional file Additional file 1). Four of the motif clusters are generally in consistence with the modules identified in this study (Table 2), including the flagellar-motor module (module 15), the osmoregulated porin gene module (13), the oxidative stress response module (2) and the methionine biosynthesis module (9). The third feed forward cluster found by Dorbin et al. [9] comprises genes of nitrogen regulation and formate regulon. They are found in two separate modules (14 and 17 respectively) in this work. In contrast to the good agreement for the five motif clusters, the other two clusters include genes belonging to many different modules. For example, the CRP cluster (see Additional file 1) consists of genes for usage of different carbon sources such as arabinose (module 5), carnitine (7), fucose (33), maltose (21), galactose (26) and mannose (18). The reason for this discrepancy is that each of the two clusters contains a global regulator (*FNR *and *CRP *respectively) which regulates genes with various functions. We further investigated the distribution of the 42 FF loops in the hierarchical structure and find that 32 of them contain one of the ten global regulators. Because modules are defined as subsets of genes with closely related functions, while global regulators tend to regulate functionally far related genes, clusters formed from network motifs which contain global regulators are not proper candidates for modules. For the four consistent motif clusters, three of them are formed from the ten FF loops that do not contain global regulators. Cluster four (osmoregulated porin gene) contains the global regulators *IHF *and *OmpR*. As shown in Fig. 1B, these two global regulators also regulate genes with flagellar motility function (module 15) and many other genes with different functions. Therefore, these two regulators cannot be properly placed in one module though most of the other genes in the cluster are functionally related. We also calculated the bi-fan motifs and find that 180 of the 209 bi-fan motifs contain global regulators. Among them 130 bi-fan motifs contain two global regulators. This means that two target operons would be coregulated by two global regulators. The fact that most network motifs contain global regulators which regulate functionally far related operons indicates that motifs cannot be regarded as elementary building blocks of functional modules because global regulators should not belong to any module with specific functions.

## Conclusions

The *E. coli *transcriptional regulatory network presently known possesses a multi-layer hierarchical structure with no feedback regulation at transcription level. Regulators in the top layers of the hierarchical structure can be considered as global regulators that often act together with local regulators to regulate genes in the bottom layer. Based on the hierarchical structure a new decomposition method is proposed which can be used to identify functional modules in the network. Analysis of operon composition of the two well-known network motifs (feed forward loop and bi-fan motif) and their distribution in the hierarchical structure suggests that they are not elementary building blocks of functional modules in the transcriptional regulatory network of *E. coli*.

## Methods

### Network reconstruction and representation

The original transcriptional regulatory database of *E. coli *was obtained from the website of Alon's research group[34]. This database is mainly based on the RegulonDB [5] and complemented by Shen-Orr et al [7]. We removed three operons (*gatR_1*, *rcsA *and *rotA*) because they are either the same with another operon or inside another operon. *GatR_1 *has been merged with *gatR_2 *in the updated annotation of *E. coli *genome in the database EcoGene [33]. *RcsA *is part of the *rcsAB *operon, while *rotA *is the same with *ppiA*. Another operon, *nycA*, was not found in any *E. coli *genome database. We searched the original literature [35] for this gene from the database obtained from Shen-Orr et al [7] and could still not find it. Therefore, we removed the *nycA *operon from the network. There are also six operons (*emrRAB*, *gatYZABCDR*, *hipBA*, *idnDOTR*, *moaABCDE *and *mtlADR*) in the network that are only autoregulated and hence do not connected with other operons. Therefore, we ignored these operons as well when analyzing the network connectivity structure. The resulting network consists of 413 nodes (operons) and 576 directed links (regulatory relationships). The 54 autoregulatory relationships in the network are represented as loops in the graph.

### Network structure analysis

Calculations for the network structure analysis were carried out by using the software Pajek [31]. The number of directly regulated operons of a regulator gene equals to its output degree, while the total number of directly and indirectly regulated operons equals to its output domain. The connected components were found by calculating the weakly connected components (the direction ignored because the regulatory network is an acyclic directed graph).

### Network motif calculation

From the hierarchical structure, feed forward loops are easily found by searching for all the fully connected triads which are located in different regulatory layers (not necessary to be three nearby layers). Bi-fan motifs are searched by using the subgraph searching algorithm in Pajek [31].

## Authors' contributions

HWM performed the analyses and drafted the manuscript. JB contributed to the concept and promoted the work. APZ is the project leader. He contributed to the concept, supervised the study and was involved in writing the manuscript. All authors have read and approved the final manuscript.

## Supplementary Material

Additional File 1 Supplenmentary table 1: Network motifs and motif clusters in the *E. coli *transcriptional regulatory network. Supplenmentary fig 1: Modules in the hierarchical structure of the *E. coli *transcriptional regulatory network. Click here for file
