# Supplementary material for: Hierarchical structure and modules in the Escherichia coli transcriptional regulatory network revealed by a new top-down approach
Source: BMC Bioinformatics. 2004 Dec 16;5:199. doi: 10.1186/1471-2105-5-199 (PMC544888; doi:10.1186/1471-2105-5-199)
Supplement: Additional File 1 — Supplenmentary table 1: Network motifs and motif clusters in the E. coli transcriptional regulatory network. Supplenmentary fig 1: Modules in the hierarchical structure of the E. coli transcriptional regulatory network. [file 1471-2105-5-199-S1.doc]

## Table 1: The 42 feed forward loops in the *E. coli* transcriptional regulatory network

| Top regulator | Mid regulator | Regulated operon | Motif cluster |
| --- | --- | --- | --- |
| flhDC | fliAZY | flgBCDEFGHIJ | 1 |
| flhDC | fliAZY | flhBAE | 1 |
| flhDC | fliAZY | fliE | 1 |
| flhDC | fliAZY | fliFGHIJK | 1 |
| flhDC | fliAZY | fliLMNOPQR | 1 |
| hns | flhDC | fliAZY | 1 |
| fnr | arcA | cydAB | 2 |
| fnr | arcA | cyoABCDE | 2 |
| fnr | arcA | focA_pflB | 2 |
| fnr | arcA | glpACB | 2 |
| fnr | arcA | icdA | 2 |
| fnr | arcA | nuoABCEFGHIJKLMN | 2 |
| fnr | arcA | sdhCDAB_b0725_sucABCD | 2 |
| arcA | appY | appCBA | 2 |
| rpoN | glnALG | nac | 3 |
| rpoN | glnALG | glnHPQ | 3 |
| rpoN | fhlA | fdhF | 3 |
| rpoN | fhlA | hycABCDEFGH | 3 |
| ihf | ompR_envZ | ompC | 4 |
| ihf | ompR_envZ | ompF | 4 |
| ompR_envZ | csgDEFG | csgBA | 4 |
| rob | marRAB | fumC | 5 |
| rob | marRAB | nfo | 5 |
| rob | marRAB | sodA | 5 |
| rob | marRAB | zwf | 5 |
| metJ | metR | metA | 6 |
| crp | fur | cirA | 7 |
| crp | melR | melAB | 7 |
| crp | araC | araBAD | 7 |
| crp | araC | araE | 7 |
| crp | araC | araFGH | 7 |
| crp | araC | araJ | 7 |
| crp | malT | malEFG | 7 |
| crp | malT | malK_lamB_malM | 7 |
| crp | malT | malS | 7 |
| crp | nagBACD | manXYZ | 7 |
| crp | nagBACD | nagE | 7 |
| crp | malI | malXY | 7 |
| crp | fucPIKUR | fucAO | 7 |
| crp | galS | mglBAC | 7 |
| crp | caiF | caiTABCDE | 7 |
| crp | caiF | fixABCX | 7 |

**Figure 1 - The modules on the hierarchical structure of *E. coli* transcriptional regulatory network.** Different node colors show different modules. The black nodes are global regulators.

**
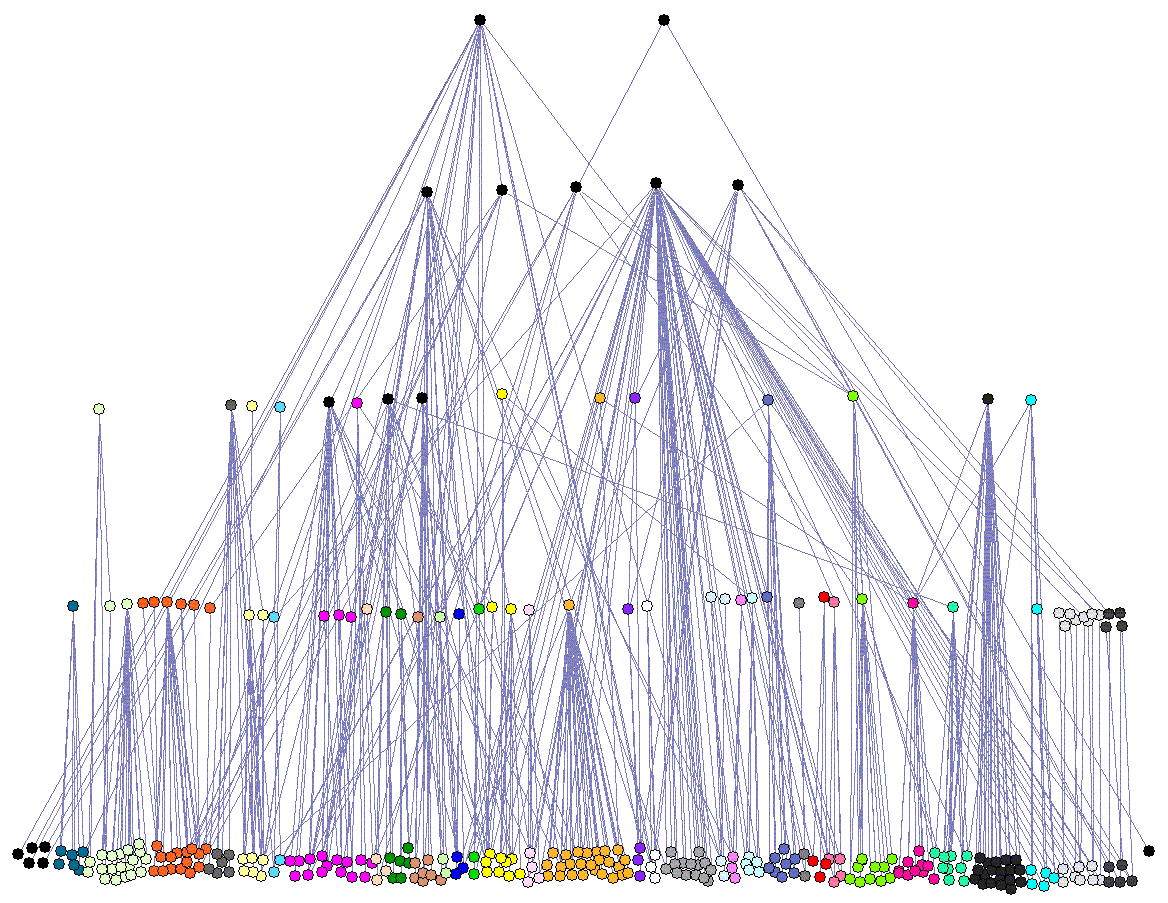
**
